# Thermodynamic Modeling of the Amorphous Solid Dispersion-Water Interfacial Layer and Its Impact on the Release Mechanism

**DOI:** 10.3390/pharmaceutics15051539

**Published:** 2023-05-19

**Authors:** Stefanie Dohrn, Samuel O. Kyeremateng, Esther Bochmann, Ekaterina Sobich, Andrea Wahl, Bernd Liepold, Gabriele Sadowski, Matthias Degenhardt

**Affiliations:** 1 AbbVie Deutschland GmbH & Co. KG, Global Pharmaceutical R&D, Knollstraße, D-67061 Ludwigshafen am Rhein, Germany; 2 Laboratory of Thermodynamics, Department of Chemical and Biochemical Engineering, TU Dortmund University, Emil-Figge-Str. 70, D-44227 Dortmund, Germany

**Keywords:** amorphous solid dispersion (ASD), drug release, phase behavior, escape glass transition (eGT), liquid-liquid phase separation (LLPS), PC-SAFT

## Abstract

During the dissolution of amorphous solid dispersion (ASD) formulations, the gel layer that forms at the ASD/water interface strongly dictates the release of the active pharmaceutical ingredient (API) and, hence, the dissolution performance. Several studies have demonstrated that the switch of the gel layer from eroding to non-eroding behavior is API-specific and drug-load (DL)-dependent. This study systematically classifies the ASD release mechanisms and relates them to the phenomenon of the loss of release (LoR). The latter is thermodynamically explained and predicted via a modeled ternary phase diagram of API, polymer, and water, and is then used to describe the ASD/water interfacial layers (below and above the glass transition). To this end, the ternary phase behavior of the APIs, naproxen, and venetoclax with the polymer poly(vinylpyrrolidone-co-vinyl acetate) (PVPVA64) and water was modeled using the perturbed-chain statistical associating fluid theory (PC-SAFT). The glass transition was modeled using the Gordon–Taylor equation. The DL-dependent LoR was found to be caused by API crystallization or liquid-liquid phase separation (LLPS) at the ASD/water interface. If crystallization occurs, it was found that API and polymer release was impeded above a threshold DL at which the APIs crystallized directly at the ASD interface. If LLPS occurs, an API-rich phase and a polymer-rich phase are formed. Above a threshold DL, the less mobile and hydrophobic API-rich phase accumulates at the interface which prevents API release. LLPS is further influenced by the composition and glass transition temperature of the evolving phases and was investigated at 37 °C and 50 °C regarding impact of temperature of. The modeling results and LoR predictions were experimentally validated by means of dissolution experiments, microscopy, Raman spectroscopy, and size exclusion chromatography. The experimental results were found to be in very good agreement with the predicted release mechanisms deduced from the phase diagrams. Thus, this thermodynamic modeling approach represents a powerful mechanistic tool that can be applied to classify and quantitatively predict the DL-dependent LoR release mechanism of PVPVA64-based ASDs in water.

## 1. Introduction

Active pharmaceutical ingredients (APIs) with low aqueous solubility and poor bioavailability are often formulated with a polymer to create amorphous solid dispersions (ASDs) [1,2,3]. The crystalline APIs are converted into an amorphous state and then embedded in a polymer to form an ASD [4,5]. The presence of the polymer leads to enhanced bioavailability of the APIs due to the increased apparent API solubility and improved dissolution behavior [6,7,8,9]. However, an ASD is only thermodynamically stable and will thus never crystallize from its amorphous state as long as the API concentration in the polymer does not exceed its solubility in the polymer matrix [6,10,11]. In ASDs with an API concentration above its solubility limit, crystallization can occur. However, crystallization might be kinetically hindered due to reduced API mobility in the polymer matrix, especially below the ASD glass transition temperature [12]. 

Numerous ASD formulations have been marketed in the last few decades [3,13]. Most commercial ASD-based oral drug products contain 10 to 20 wt % of drug load (DL) [14,15,16,17]. When it comes to the dissolution of the ASD into an aqueous medium, the release mechanism is affected by the polymer and API type, as well as the DL [18]. The DL of ASD is often limited because of the observed phenomenon of collapsing API release with an increasing DL. In practice, this drawback is usually mitigated by selecting a different polymer or adding an appropriate surfactant to the ASD. The phenomenon’s origin and mechanism are therefore highly relevant in ASD formulation development and have been widely investigated. Earlier, Craig [19] hypothetically proposed release mechanism for ASD dissolution process as (a) carrier-controlled dissolution, whereby the API forms a concentrated layer with the polymer prior to their release, or (b) API-controlled dissolution, whereby the API is released effectively into the dissolution medium as intact particles. The author further argued that, depending on the solubility of the APIs in the concentrated solution of the polymer, one of these two release mechanisms is predominant [19]. Experimentally, Treacher et al. [20,21] investigated the DL-dependent dissolution profiles of felodipine/vinylpyrrolidone-co-vinyl acetate (PVPVA64) ASDs and systematically observed a collapse of felodipine dissolution with an increasing DL. Later, Taylor and coauthors [22,23,24,25] defined this phenomenon as the “limit of congruency” (LoC), which refers to the highest DL until the congruent release of the API and the polymer is observed. Based on experimental observations, they explained that beyond the LoC, the kinetics of amorphous phase separation (AAPS) at the ASD surface was faster than the dissolution rate of the ASD, thus leading to a slowly dissolving API-rich phase from the ASD surface, while the polymer preferentially went into solution [22]. Saboo et al. [24] experimentally studied the impact of the APIs’ physicochemical properties, including hydrogen bonding and the glass transition on the release mechanism of ASDs. Yang et al. [26] used confocal fluorescence microscope images of ritonavir/PVPVA64 ASDs to show the underlying role of the API-polymer phase separation on the release mechanism of ASDs. Spectroscopic imaging techniques such as FT-IR [27,28], Raman spectroscopy [29,30], and magnetic resonance [31] have been employed to investigate the interfacial behavior of ASDs during dissolution. Similarly, microscopy imaging techniques, including confocal fluorescence microscopy [26], scanning electron microscopy (SEM) [32], and transmission electron microscopy (TEM) [33] have also been used for these investigations. More recently, in silico molecular dynamics simulations have been employed to understand the impact of the polymer type and interfacial behavior of ASDs during dissolution [34].

Bochmann et al. microscopically investigated the ASD/water interfacial behavior of several PVPVA64-based ASDs [18]. The authors systematically studied 19 different APIs and the DL-dependent behavior of their ASD/water interface during dissolution. For each API, a specific DL threshold was found above which the ASD/water interfacial gel layer switches from an eroding to a non-eroding front due to the crystallization of the API at the interface or the formation of a compact gel layer that swells rather than erodes [18]. Depending on the API, the DL threshold ranged between 5 wt % and 50 wt %. Interestingly, the DL thresholds of 15 wt % and 32.5 wt % observed by Bochmann et al. [26] for indomethacin-PVPVA64 and ritonavir-PVPVA64 ASDs, respectively, correspond very well with the LoC values reported by Taylor and coauthors [24]. This suggests that the ASD/water interfacial behavior strongly influences the API release mechanism during ASD dissolution. 

Whereas the effects of collapsing API release with increasing DL have been generally observed, extensively investigated, and discussed in the literature, the underpinning thermodynamic principles of the phenomenon are poorly understood and, hence, are less widely reported [8,22,28,35]. Recently, Han et al. [36], using the Flory–Huggins theory, applied an API/polymer/water ternary phase diagram to semi-quantitatively describe the impact of phase separation and morphology on the ASD release mechanism. The approach of using ternary phase diagrams to quantitatively predict and describe the phase behavior of ASDs in a solvent based on the perturbed-chain statistical associating fluid theory (PC-SAFT) [37] was first published by Luebbert et al. [38] and then further applied to solvent-based ASD manufacturing by Dohrn et al. [39,40,41]. Recently, Krummnow et al. [42] used a ternary phase diagram, derived from PC-SAFT and the Kwei equation [43], to quantitatively predict liquid-liquid phase separation (LLPS) and successfully proposed the release mechanism of ritonavir/PVPVA64 ASDs into the dissolution medium. Although these publications provided thermodynamic insights, a generalized explanation of the phase behavior within the ASD/water interfacial layer had not yet been developed. 

Hence, this work focuses on addressing the missing thermodynamic modeling of the ASD/water interfacial layer as several of the publications have shown that the interfacial layer behavior subsequently dictates the ASD release mechanism. For this purpose, we chose two APIs, naproxen and venetoclax, with extremely different physicochemical properties in terms of their crystallization propensity and the glass-transition temperature (*T*_g_) of the amorphous phase. While naproxen has a low *T*_g_ of −8 °C and crystallizes rapidly from the amorphous phase [43], venetoclax on the other hand has a very high *T*_g_ of 120 °C and does not readily crystallize from the amorphous phase. The ternary API/polymer/water phase diagrams of naproxen-PVPVA64 and venetoclax-PVPVA64 ASDs with water were modeled using PC-SAFT to define the APIs’ solubility and LLPS, which is also known colloquially as AAPS. The glass transition was calculated using the Gordon–Taylor equation. The ternary phase diagrams were applied to quantitatively predict the limit of release (LoR) of the ASDs at 37 °C and were experimentally validated with microscopic imaging, Raman spectroscopy, size exclusion chromatography (SEC), and USP II dissolution tests. Furthermore, the phase diagrams were predicted at 50 °C and then experimentally validated to understand the impact of temperature on the ASD release mechanism.

## 2. Modeling

### 2.1. Solid-Liquid Equilibrium

The equilibrium between the pure solid (crystalline) phase and the liquid (amorphous) phase was considered for calculating the mole fraction solubility $x_{i}^{L}$ of a crystalline API i in a solvent (here, water) or a polymer (here, PVPVA64) and the mixtures thereof, according to Equation (1):

(1) $$x_{i}^{L}=\frac{1}{\gamma_{i}^{L}}exp\left[-\frac{\Delta h_{i}^{SL}}{RT}\left(1-\frac{T}{T_{i}^{SL}}\right)-\frac{\Delta c_{p,i}^{SL}}{R}\left(\ln\left(\frac{T_{i}^{SL}}{T}\right)-\frac{T_{i}^{SL}}{T}+1\right)\right]$$

where T is the temperature, and R is the universal gas constant. The melting properties of component i, namely, the melting temperature $T_{i}^{SL}$, the melting enthalpy $\Delta h_{i}^{SL}$ and the difference between solid and liquid heat capacities $\Delta c_{p,i}^{SL}$ of the APIs (Table 1), were measured for venetoclax in this work and used figures taken from the literature for naproxen. The activity coefficient $\gamma_{i}^{L}$ of component i in the liquid phase accounts for deviations from an ideal mixture and was determined using PC-SAFT (Section 2.4).

### 2.2. Liquid-Liquid Equilibrium

The equilibrium compositions of two coexisting liquid (amorphous) phases, L1 and L2, were determined by simultaneously solving Equation (2) for every component *i*:

(2) $$x_{i}^{L1}\gamma_{i}^{L1}=x_{i}^{L2}\gamma_{i}^{L2}$$

where $x_{i}^{L1}$ and $x_{i}^{L2}$ are the mole fractions of component *i* in phase L1 and phase L2, respectively. The calculated mole fractions were converted into mass fractions in the phase diagrams. The activity coefficients $\gamma_{i}^{L1}$ and $\gamma_{i}^{L2}$ of component i in phase L1 and phase L2 account for the strong deviations in the interactions compared to an ideal mixture, as calculated with PC-SAFT.

### 2.3. Glass-Transition

The glass transition of the API/polymer/water mixtures was predicted using the Gordon–Taylor equation [44] (Equation (3)).

(3) $$T_{g}=\frac{\sum_{i}K_{i}w_{i}T_{g,i}}{\sum_{i}K_{i}w_{i}}$$

Here, *w_i_* are the mass fractions of *i* = polymer, API, and water, respectively. *T*_g*,i*_*,* is the glass-transition temperature of each pure component. The pure-component glass-transition temperatures, *T*_g_, and true densities, *ρ*, were measured in this work for venetoclax or were taken from the literature and are listed in Table 2. The Gordon–Taylor interaction parameters, *K*, were predicted using the Simha–Boyer rule [45], according to Equation (4):

(4) $$K_{i,PVPVA64}=\frac{\rho_{PVPVA64}T_{g,PVPVA64}}{\rho_{i}T_{g,i}}.$$

### 2.4. PC-SAFT

The activity coefficients used in this work were calculated using PC-SAFT [52] from the residual Helmholtz energy, $a^{res}$, calculated via summing up the specific molecular contributions caused by repulsion (hard chain *a*^hc^), attraction (dispersion *a*^disp^), and association (*a*^assoc^), according to Equation (5):

(5) $$a^{res}=a^{hc}+a^{disp}+a^{assoc}$$

Each molecule has a defined number of segments ($m_{i}^{seg}$*)* with a segment diameter of $\sigma_{i}$ and a dispersion energy parameter of ${(u}_{i}/k_{B})$. Hydrogen-bond forming molecules are characterized by the association sites, *N_i_*^assoc^, the association-energy parameter $\left(\varepsilon^{A_{i}B_{i}}/k_{B}\right)$, and the association-volume parameter, $\kappa^{A_{i}B_{i}}$. $k_{B}$ is the Boltzmann constant. The pure-component parameters of venetoclax were derived in this work (Figure S1 and Table S1 in the Supplementary Materials), and the parameters of naproxen, PVPVA64, and water were taken from the literature (Table 3).

The combined rules of Berthelot [55] (Equation (6)) and Lorentz [56] (Equation (7)) were applied to determine the segment diameter, $\sigma_{ij},$ and the dispersion energy, $u_{ij},$ in the mixtures of components i and j:

(6) $$\sigma_{ij}=\frac{1}{2}\left(\sigma_{i}+\sigma_{j}\right)$$

(7) $$u_{ij}=\sqrt{u_{i}u_{j}}\left(1-k_{ij}\right)$$

To calculate the association energy and the association volume in the mixtures of components i and j, the combined rules of Wolbach and Sandler [57] (Equations (8) and (9)) were used:

(8) $$\varepsilon^{A_{i}B_{j}}=\frac{1}{2}\left(\varepsilon^{A_{i}B_{i}}+\varepsilon^{A_{j}B_{j}}\right)$$

(9) $$\kappa^{A_{i}B_{j}}=\sqrt{\kappa^{A_{i}B_{i}}\kappa^{A_{j}B_{j}}}{\left(\frac{\sqrt{\sigma_{i}\sigma_{j}}}{\frac{1}{2}\left(\sigma_{i}+\sigma_{j}\right)}\right)}^{3}.$$

The binary interaction parameter, $k_{ij,}$ corrects for deviations from the geometric mean of the dispersion energies of the pure components and might depend on temperature, as expressed in Equation (10):

(10) $$k_{ij}=k_{ij,m}T+k_{ij,b}$$

The coefficients $k_{ij,m}$ and $k_{ij,b}$ were determined in this work via fitting to the data of binary mixtures (Table S1 and Figure S1 in the Supplementary Materials) and were taken from the literature. Table 4 lists all the interaction parameters used in this work.

## 3. Materials and Methods

### 3.1. Materials

Naproxen was supplied by Cayman Chemical (Ann Arbor, MI, USA), and venetoclax was obtained from AbbVie Inc. (Chicago, IL, USA). Vinylpyrrolidone-vinyl acetate copolymer (PVPVA64, Kollidon^®^ VA 64) was purchased from BASF SE (Ludwigshafen, Germany). Methanol, water (LS-MS Grade), trifluoroacetic acid, and acetonitrile were purchased from Merck KGaA (Darmstadt, Germany). For the dissolution experiments, purified water without buffer was used. Unbuffered water was chosen because the modeling calculations in Section 2 were performed using water without any buffer system. 

### 3.2. Methods

#### 3.2.1. Preparation of Amorphous Solid Dispersion Discs for the Microscopic Erosion Time Test (METT)

The ASDs used in the METT experiments were based on cryo-milled physical blends, which were subsequently melted and shaped using a vacuum compression molding (VCM) tool (MeltPrep GmbH, Graz, Austria) [18]. For the cryo-milling, approximately 1 g of each API/PVPVA64 physical blend was loaded into 10 mL stainless steel chambers with a 15 mm stainless steel ball. The chambers were put into liquid nitrogen and subsequently milled in an MM400 device (Retsch GmbH, Haan, Germany) at 30 Hz for 30 s. To prepare disc-shaped ASDs, approximately 50 mg of the cryo-milled blend was loaded into the VCM tool with a 10 mm diameter disc geometry and then annealed for 20 min. Annealing temperatures of 180 °C or 150–160 °C for venetoclax- or naproxen-containing samples, respectively, were applied. The absence of crystalline residuals in the prepared discs was confirmed using polarized light microscopy.

#### 3.2.2. Microscopic Erosion Time Test (METT)

The erosion behavior of the prepared ASD discs (see Section 3.2.1) was analyzed by means of METT [18]. The test was performed with a Keyence VH-X digital microscope (Keyence Deutschland GmbH, Neu-Isenburg, Germany) on which a water-tempered copper plate was mounted. The copper plate was pre-heated to either 37 °C or 50 °C by a Thermostat Haake A10 (Thermo Fisher Scientific, Karlsruhe, Germany). On the copper plate, the ASD disc was placed between an object slide and a coverslip. Pre-heated degassed demineralized water was added and then the edges of the coverslip were sealed with nail polish to prevent water evaporation. The course of the ASD erosion in water was monitored over 60 min by taking a picture every 10 min.

#### 3.2.3. Preparation and Dose Strength Calculation of Naproxen ASD Discs for Dissolution Experiments

In dissolution experiments, naproxen ASDs with drug loads of 10 wt %, 20 wt %, and 30 wt % were targeted. To enable the preparation of 20-millimeter diameter disc-shaped ASDs and a dose strength for non-sink conditions, the following calculations were considered:

1. The solubility of naproxen in water was calculated to be 0.429 g/L by Percepta (ACD/Labs Release 2020.1.2 (Build 3382, 18 June 2020), Advanced Chemistry Development Inc., Toronto, ON, Canada).
2. A dose strength of 386 mg would be required to achieve a 10-fold higher maximum concentration than the estimated naproxen solubility in a 900 mL dissolution medium.
3. The dose strength of 386 mg led to a target weight of 3.86 g, 1.93 g, and 1.29 g for 10 wt %, 20 wt %, and 30 wt % DL ASD discs, respectively. This translates into 20 mm ASD discs of 10.4 mm, 5.2 mm, and 3.5 mm in height, respectively.

Physical blends of naproxen and PVPVA64 were prepared, hot-melt extruded, milled, and subsequently shaped by the VCM tool into ASD discs. Physical blends with either 10 wt %, 20 wt %, or 30 wt % of naproxen in PVPVA64 were blended for 3 min in a Turbula mixer at 45 rpm (Willy A Bachofen AG, Muttenz, Switzerland), manually sieved through an 800 µm sieve, and were again blended for another 3 min.

For hot-melt extrusion, a 9 mm ZE9 twin-screw extruder (ThreeTec GmbH, Seon, Switzerland) with a 2 mm die was used. The extruder was equipped with a ZD 5 FB-C-1M-50 feeder (ThreeTec GmbH, Seon, Switzerland) in volumetric feeding mode to allow a 1 g/min feed rate. The extruder barrel consisted of 6 zones heated to 20/70/95/140/140/140 °C, 20/70/90/130/130/130 °C, and 20/60/80/120/120/120 °C for the 10 wt %, 20 wt %, and 30 wt % DL physical blends, respectively. The extruder screws consisted of majorly conveying elements with 60° kneading elements, along with 60° followed by 90° kneading elements in zones 4 and 5, respectively. A screw speed of 100 rpm was applied.

The produced extrudates were milled with an IKA A10 basic mill (IKA-Werke GmbH & Co., Staufen, Germany) in 10-second cycles, then manually sieved through a 355 µm sieve. The milled extrudate was further processed by using a VCM with a 20-millimeter diameter disc geometry. Samples were annealed for 7 min at a temperature corresponding to the HME extrusion temperature at the last barrel section before the die (140 °C, 130 °C, and 120 °C for a DL of 10 wt %, 20 wt %, and 30 wt %, respectively). The absence of crystalline residues in the prepared ASD discs was verified using polarized light microscopy.

#### 3.2.4. Dissolution Experiments

The dissolution of the naproxen ASD discs was performed in demineralized water at 37 °C. The dissolution tests were run on a USP 2 apparatus (Hanson Classic 6, Hanson Research Corporation, Chatsworth, LA, USA) with its corresponding paddle for 1 L vessels (the distance from the paddle to the bottom was adjusted with a calibration ball to 25 mm). The paddle rotation speed was set to 75 rpm and the dissolution medium volume was 900 mL. During the dissolution experiments, samples were taken automatically via autosampler (Maximizer TM Hanson Research Corporation, Chatsworth, LA, USA) at 15 min, 30 min, 45 min, 1 h, 1.5 h, and 2 h intervals. The sampling volume withdrawn by the autosampler was 1.5 mL and the API and polymer contents were determined via reverse-phase (RP) HPLC and size exclusion chromatography (SEC), respectively (see Section 3.2.5). For each formulation, the dissolution test was performed in triplicate.

#### 3.2.5. Liquid Chromatography Analysis

The dissolution samples described in Section 3.2.4 were dried in the vacuum chamber (40 °C; 70 mbar; 72 h) to evaporate off the water. The dried samples were then dissolved in 1.5 mL methanol. For samples originating from the 10 wt % and 20 wt % DL dissolution experiments, the methanol solutions were further diluted 20-fold and 2-fold for the RP-HPLC and SEC analysis, respectively. A set of standards in the concentration range of 0.59 to 29.7 μg/mL was prepared for naproxen content determination. The PVPVA64 calibration standards were prepared in the concentration range of 7.5 to 3000 µg/mL c. Methanol was used as a solvent for the calibration standards. The calibration standards and dissolution samples were centrifuged (4 °C; 21,000 rpm; 5 min) before the RP-HPLC and SEC analyses. RP-HPLC was performed on an advanced polymer chromatography (APC) system equipped with a quaternary solvent manager (p-QSM) and an ACQUITY PDA detector (Waters Corp, Milford, MA, USA) using a Kinetex C18 2.6 µm 50 × 2.1 mm 100 Å column (Phenomenex, Torrance, CA, USA). The column temperature was maintained at 60 °C. The injection volume was 2 µL and the flow rate was 1.2 mL/min with mobile phase A: 0.1% (*v*/*v*) trifluoroacetic acid in water, and mobile Phase B: acetonitrile. The gradient started with a linear increase in acetonitrile from 5% to 95% within 2.33 min, holding for 1 min, then a decrease to 5% acetonitrile. The total run time of the gradient was 3.65 min. Naproxen was detected at 232 nm and showed a retention time of 1.4 min. The samples were measured in triplicate.

SEC analysis was also performed on the APC using a set of two ACQUITY APC XT 45 Å 1.7 mm 4.6 mm × 150 mm columns (Waters, Milford, MA, USA) with an 0.2 µm in-line pre-filter. The column temperature was set to 55 °C. Measurements were taken under isocratic conditions with methanol as the mobile phase, at a flow rate of 0.9 mL/min. The run time of each measurement was 6 min. The polymer signal was detected at 203 nm and had a retention time of 1.8 min. The samples were measured in triplicate.

#### 3.2.6. SEC Analysis of the ASD and the Liquid Phase in METT Experiments

After the venetoclax ASD METT experiments (see Section 3.2.3), samples were collected from the ASD disc core and the aqueous phase. The aqueous phase samples were dried overnight in the vacuum chamber (40 °C; 70 mbar) to remove the water. The ASD disc core and the dried aqueous phase samples were separately dissolved in THF. The solutions were adjusted to 2 mg/mL (for 0.5 wt % and 1 wt % DL of ASD) or 1 mg/mL (for 2.5 wt % DL of ASD)). All samples were centrifuged at 21,000 rcf and 4 °C for 5 min. The composition of the dried aqueous phase was determined, relative to the ASD disc core composition.

The SEC analysis was performed on the APC using a set of one ACQUITY APC XT 200 Å 2.5 mm 4.6 mm × 150 mm and two ACQUITY APC XT 45 Å 1.7 mm 4.6 mm × 150 mm columns (Waters, Milford, MA, USA), with an 0.2 µm in-line pre-filter. The columns’ temperature was set at 40 °C. The separation was isocratic, with THF as a mobile phase, at a flow rate of 0.9 mL/min. The polymer and venetoclax signals were detected at 210 nm and 300 nm, respectively. The samples were measured in triplicate.

#### 3.2.7. Raman Spectroscopy

An ASD disc with 2.5 wt % DL of venetoclax, before and after exposure to de-ionized water at 37 °C for 4 h and vacuum-dried at 25 °C, was analyzed with a Raman microscope (HORIBA Labram HR Evolution, Oberursel, Germany). A near-IR (633 nm) laser was employed to illuminate the samples. Spectra were collected using a 100×/0.90 objective and a 100 µm confocal pinhole. A 600 line·mm^−1^ rotatable diffraction grating was used, set at 5 s of acquisition time and 8 accumulations. Spectra were collected in the range from 250 to 3750 cm^−1^.

## 4. Results and Discussion

### 4.1. Modeling Approach for the ASD/Water Interface

As soon as the surface of an ASD encounters water, several interplay processes, including water sorption, phase separation, crystallization, and the viscosity of the formed (gel) layer, determine the ASD/water interfacial behavior and, subsequently, the dissolution performance of the formulation. The physicochemical properties of the APIs and the polymer, their interactions in the ASD, and, ultimately, their interactions with water determine the thermodynamics and kinetics of these processes. Previous works by Sadowski and coauthors demonstrated that phase transformations (crystallization and phase separation) associated with ASD/water can be thermodynamically modeled quantitatively [11,38,39,53,58,59,60,61]. The kinetics aspect is strongly influenced by the glass transition behavior of the system and the individual components [12]. The interplay between phase transformations and glass transition during the ASD dissolution can be understood through the API/polymer/water ternary phase, coupled with the glass transition. 

**Figure 1:**
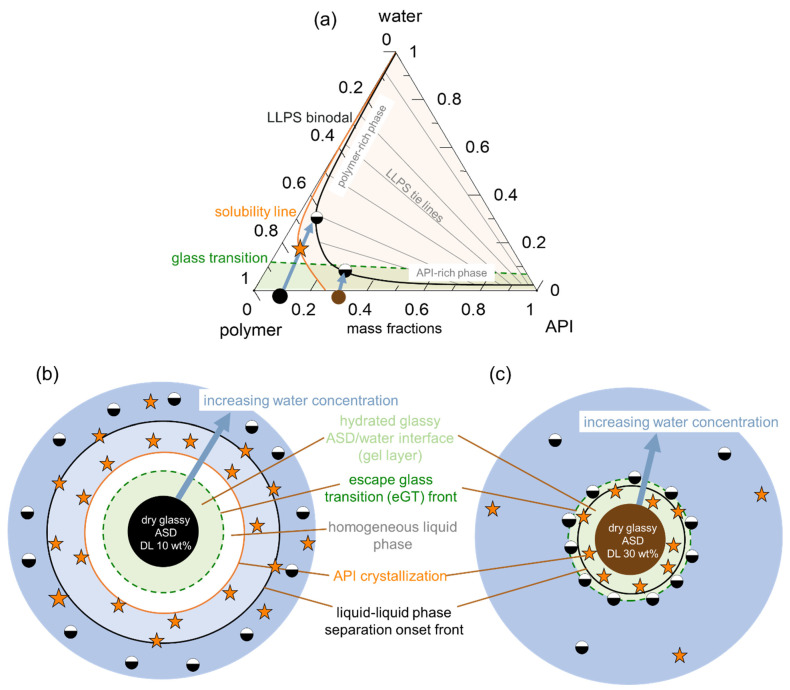
(**a**) Schematic ternary phase diagram of an API/polymer/water system, showing the solubility line (orange), LLPS boundary (black line), tie lines (gray lines), and the glass transition (green dashed line). The symbols refer to the dry DL 10 wt % ASD (black circle), the dry 30 wt % DL ASD (brown circle), API crystals (orange stars), and LLPS (half-filled circles). The arrows indicate the hydration pathway through the ASD/water interface, starting from the dry ASD towards increasing water concentration, as depicted in (**b**,**c**). The latter are schemes of the ASDs in contact with water and the surrounding interface layers (following the blue arrow direction in (**a**)) depicting the first interfacial region below the glass transition (green region), the interfacial region after the “escaping the glass transition” (eGT) (green dashed line), followed by a region of homogeneous liquid (white region), beyond which API crystallization (orange line) and LLPS (black line) occur.

Figure 1 schematically shows the ternary phase diagram (Figure 1a) of an API/polymer/water system and the hydration pathway of the ASD as it transitions into the bulk aqueous medium during dissolution, as exemplified for ASDs with 10 wt % (Figure 1b) and 30 wt % of DL (Figure 1c). The ternary phase depicts the regions of API crystallization or LLPS that form when the ASD is exposed to water. Depending on the concentration of the ternary mixture, the driving force(s) for equilibrium determines what phase changes occur, e.g., either crystallization in API-supersaturated regions or LLPS, or both. Furthermore, at concentrations below the glass transition (the green region in Figure 1a), the mobility of the system is reduced, which can kinetically hinder phase changes.

Considering the hypothetical 10 wt % DL ASD, on contact with water, the surface of the dry ASD immediately starts absorbing water along the hydration pathway (blue arrow). Simultaneously, the glass transition temperature (*T*_g_) at the ASD interface decreases, due to plasticization by the imbibed water molecules, thus forming a so-called gel layer at the surface of the ASD. The plasticization of the surface layer continues as more water is absorbed until the *T*_g_ reaches the temperature of the dissolution medium. At this point, cooperative motion and the disentanglement of the polymer chains occur, initiating the dissolution of the ASD surface into the aqueous phase. We term the *T*_g_ of the ASD surface at this point “escape glass transition” (eGT), as indicated by the dashed green line in Figure 1a. Thus, the calculated eGT line represents the API/polymer/water compositions, wherein the *T*_g_ of the system is equivalent to the temperature of the dissolution medium. 

As the API and polymer continuously evolve from the interface into the aqueous phase along the hydration pathway, their concentration in the solution increases until the API’s maximum solubility is reached (orange line), beyond which the API may start to crystallize out of the supersaturated bulk solution. A further increase in API and polymer concentrations results in the solution entering the binodal region, wherein the system is prone to LLPS, leading to polymer-rich and API-rich phases along the tie lines shown in Figure 1a. In Figure 1b, the consequential impact of the hydration pathway on the 10 wt % DL ASD during dissolution is schematically depicted as an ASD disc in contact with water. Importantly, crystallization or LLPS is not expected to happen in the gel layer at the ASD/water interface since these phase transformations occur beyond the eGT. Similarly, following the hydration pathway of a hypothetical 30 wt % DL of ASD in Figure 1a shows that the dry ASD is already in the supersaturated region to the right of the solubility line (orange line). Moreover, the hydration pathway encounters the LLPS region before reaching the eGT. Thus, contrary to the 10 wt % DL ASD, crystallization and/or LLPS is expected to occur in the gel layer at the ASD/water interface, as schematically depicted in the ASD disc in Figure 1c. 

**Figure 2:**
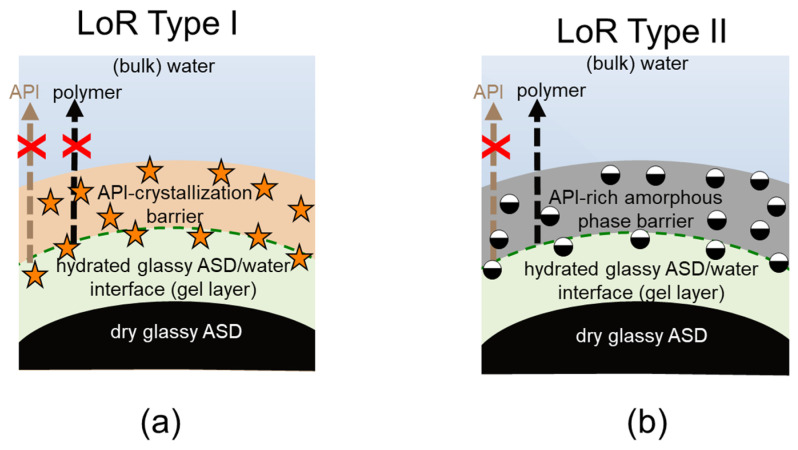
Schematics of LoR Type I and Type II for ASD/water interfacial layers, resulting in LoR (passivation) due to the crystallized API barrier formed at the surface (**a**), and due to the amorphous API barrier triggered by liquid-liquid phase separation at the surface (**b**).

Obviously, the gel layer thus formed is not static and can be perturbed by hydrodynamics during the dissolution test of an ASD formulation. However, we postulate that it is critical to know the dominant thermodynamic driving forces at the ASD/water interface, especially in the first boundary layer below eGT, which is often referred to as the gel layer, in order to understand whether a phase change might or might not occur. Phase changes in the gel layer can lead to the passivation of the interface, resulting in the loss of release (LoR) of the API and polymer. The LoR mechanism can be categorized into two types, depending on whether the passivation is primarily driven by liquid-solid transformation (crystallization), LoR Type I, or liquid-liquid (amorphous) phase separation, LoR Type II, at the interface, as schematized in Figure 2.

For the LoR mechanism Type I, the formation of an inelastic crystalline passivation layer prevents the release of both the API and the polymer. On the other hand, for LoR mechanism Type II, the development of a hydrophobic API-rich amorphous phase barrier, due to LLPS, results in the preferential release of the complementary hydrophilic polymer-rich phase into the aqueous medium. Hence, the LoR Type II mechanism results in the so-called incongruent release of the API and polymer [24]. It is also possible for both mechanisms to concurrently contribute to LoR, depending on the ternary phase diagram and crystallization propensity of the API.

To demonstrate the a priori applicability of the ternary phase diagram in predicting LoR, we chose two PVPVA64-based ASDs, formulated with either naproxen or venetoclax. For comparison purposes, having a common polymer for both formulations helps to normalize the polymer’s impact and other contributing factors to some extent in terms of the gel layer’s behavior, such as its viscosity and water-sorption kinetics.

### 4.2. LoR Type I: Naproxen/PVPVA64/Water System

**Figure 3:**
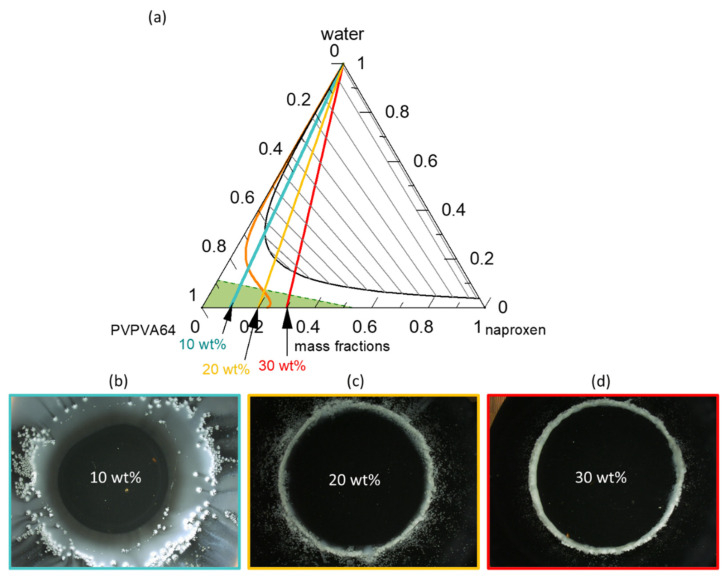
Ternary-phase diagram of naproxen/PVPVA64/water (**a**) at 37 °C and 0.1 MPa. The green dashed line represents the glass transition, the orange line represents the solubility line, the black line represents the LLPS boundary (binodal line) with gray tie lines, and the blue, yellow, and red straight lines represent the hydration pathways of 10 wt %, 20 wt %, and 30 wt % DL, respectively; METT images after 40 min of dissolution of a (**b**) 10 wt %, (**c**) 20 wt %, and (**d**) 30 wt % DL ASD disc.

Figure 3a presents the modeled naproxen/PVPVA64/water ternary phase diagram at 37 °C; Figure 3b–d shows the METT images of the ASDs at DLs of 10 wt %, 20 wt %, and 30 wt % when in contact with water.

The solid-liquid (API solubility line) and liquid-liquid equilibria lines were modeled using PC-SAFT and the eGT line was calculated using the Gordon-Taylor equation. The phase diagram shows that in the dry state, ASDs with DL up to 23 wt % are thermodynamically stable (intersection of solubility line with the PVPVA64/naproxen axis) and thus will not crystallize. From the phase diagram, it is clear that naproxen has a greater solubility in the PVPVA64 than in water since the solubility strongly decreases with increasing water concentration in the API/polymer/water mixture. The interplay between the solubility line and eGT line along the hydration pathway is decisive in predicting the crystallization of the API at the ASD/water interface during dissolution. As explained schematically in Figure 1 for DLs below the intersection of the solubility line and the eGT line, crystallization is expected to occur in the aqueous phase away from the gel layer (e.g., in the dissolution medium). For DLs above this intersection, crystallization is expected in the gel layer, leading to the risk of LoR. Thus, based on the phase diagram, above 19 wt % DL ASD, there exists a risk of naproxen crystallization in the gel layer of the ASD because the hydration pathway intersects the solubility line before the eGT line. Moreover, the phase diagram predicts that regardless of the naproxen DL, LLPS will not occur in the gel layer but instead appear in the aqueous bulk medium since the LLPS phase transition is well above the eGT line. The readout API/water/polymer concentrations along the hydration pathway in the phase diagram for the investigated 10 wt %, 20 wt %, and 30 wt % DL naproxen ASDs are given in Table 5.

The water content at the solubility limit of the 10 wt % DL ASD is 16.3 wt %, which is more than the 9.2 wt % water content that is required to reach eGT; hence, API crystallization at high water concentrations outside the gel layer (i.e., in the dissolution bulk phase) is expected. For the 20 wt % DL ASD, the water content at the solubility limit and eGT are 6.1 wt % and 7.3 wt %, respectively, meaning that crystallization will occur in the gel layer but will be at the front, close to the aqueous bulk medium. However, for the 30 wt % DL ASD, the solubility limit is already exceeded in the dry state. Hence, crystallization will take place with the least amount of water absorbed at the interface, leading to the formation of a compact crystalline front. Thus, the consequential LoR Type I, due to crystallization in the gel layer, is expected to be more pronounced for the 30 wt % DL ASD than the 20 wt % DL ASD.

**Figure 4:**
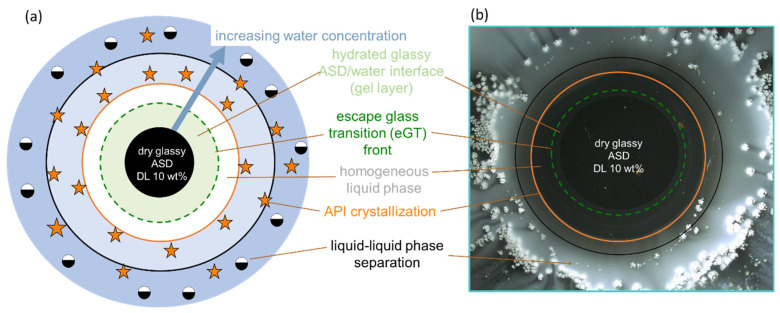
(**a**) Zoomed-in diagram of the interfacial layers of the METT images after 40 min of dissolution of the 10 wt % DL naproxen ASD (Figure 3b) and (**b**) the scheme of the ASD in contact with water and the surrounding interface layers, depicting the first interfacial region below the glass transition (green region) and the interfacial region after “escaping the glass transition” (eGT) (green dashed line), followed by a region of a homogeneous liquid (white region), beyond which API crystallization onset (orange line) and LLPS onset (black line) both occur.

To verify the predicted gel layer behavior, the dissolution of 10 wt %, 20 wt %, and 30 wt % DL naproxen ASDs discs were monitored via METT experiments. According to the classification system presented by Baird et al. [58], neat naproxen crystallizes extremely rapidly from the amorphous phase. Although the 30 wt % DL ASD in the initial dry state is thermodynamically unstable at 37 °C (Figure 3a), naproxen does not spontaneously crystallize because the API molecules are kinetically stabilized in their glassy state in PVPVA64 (the green region in Figure 3a) [39]. Figure 3b–d presents the images of the ASD discs after 1 h of contact with water at 37 °C. Videos of the evolution of the ASD disc/water interface over the period are provided in the Supplementary Materials. The METT experiment setup is a very effective technique for monitoring the gel layer since the hydrodynamic effects are minimal. As seen in Figure 3b, naproxen crystallizes as a bright white domain well beyond the surface of the 10 wt % DL ASD disc, which appears as a dark circle in the image. Moreover, it can also be noticed that a concentric white solution phase has also developed far away from the interface. The observed effect is attributed to LLPS, which is predicted to occur beyond the gel layer. Thus, the evolution of the 10 wt % naproxen ASD/water interface agrees perfectly with the ternary phase diagram prediction shown in Figure 3a. In contrast, for the naproxen ASDs with DLs of 20 wt % and 30 wt %, the white naproxen crystalline domains formed on the disc surface when in contact with the aqueous medium, as seen in Figure 3c,d, respectively, indicating crystallization in the gel layer, as was predicted. Barely perceptible differences can be observed between the crystallized interfacial layer of the two formulations. Specifically, in the 30 wt % DL ASD, the crystals compact tightly at the surface of the disc, compared to the 20 wt % DL ASD disc. The METT images are, therefore, in very good agreement with the PC-SAFT-predicted phase behavior for ASD hydration pathways (Figure 3a, Table 6). Figure 4 gives more detailed insights into the visual layer formation that occurred in the METT investigations (Figure 3b).

**Figure 5:**
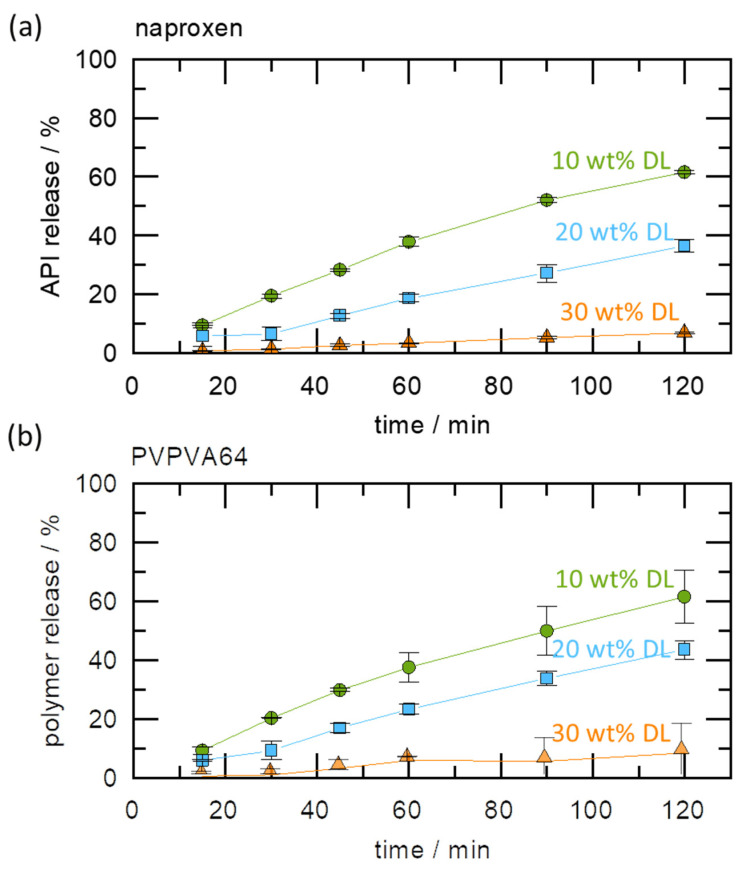
Release profiles of (**a**) naproxen and (**b**) PVPVA64 at 37 °C for naproxen/PVPVA64 ASD, with DLs of 10 wt % (green circles), 20 wt % (blue squares), and 30 wt % (orange triangles).

The impact of the gel layer crystallization on dissolution was further investigated by monitoring the release profiles of naproxen and PVPVA64 from the ASDs at 37 °C, using a USP 2 apparatus. The results are displayed in Figure 5a,b.

It can be observed that APIs and polymer release levels are highest for 10 wt % DLASD and lowest for 30 wt % DL ASD. While the former released 60% of both the API and polymer in 2 h, the latter released only 10% in the same period. As pointed out earlier, the Type I LoR mechanism triggers the formation of a solid crystalline barrier at the ASD/water interface, as seen in the METT image, which prevents the free release of both the API and the polymer. For the 20 wt % DLASD, although there is an LoR, the API and polymer release levels are significantly higher compared to the 30 wt % DL ASD. This can be explained by the fact that the crystallization barrier at the ASD/water interface is expected to be less compact and is more easily disrupted by the hydrodynamics of the dissolution experiments than for the 30 wt % DL ASD. It is worth noting that from the three ASDs, both the API and the polymer released at similar rates, the so-called congruent release, even when LoR occurred. This supports our hypothesis that API and polymer release rates are nearly equally affected when LoR is triggered by the LoR Type I mechanism. Thus, the results of the dissolution and METT experiments support and validate the quantitatively predicted release behavior, based on the modeled ternary phase diagram at 37 °C (Figure 3). 

### 4.3. LoR Type II: Venetoclax/PVPVA64/Water System

**Figure 6:**
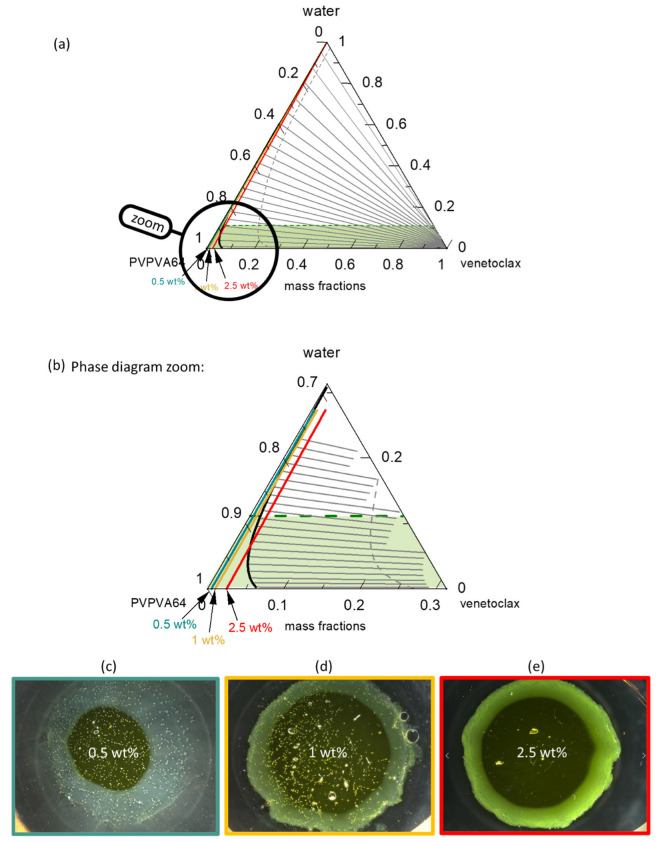
(**a**) Ternary phase diagrams of venetoclax/PVPVA64/water at 37 °C and 0.1 MPa and zoomed-in image of the ternary phase diagram in (**b**). The green dashed line represents the glass transition, the orange line represents the solubility line, the black line represents the LLPS boundary (binodal line) with gray tie lines and the gray dashed spinodal line, and the blue, yellow, and red straight lines represent the hydration pathways for 0.5 wt %, 1 wt %, and 2.5 wt % DL ASD, respectively; METT images after 30 min of dissolution of the (**c**) 0.5 wt % DL, (**d**) 1 wt % DL, and (**e**) 2.5 wt % DL ASD disc.

For an API that does not readily crystallize from the amorphous phase, as with venetoclax, the solubility line in the ternary phase diagram is less decisive for LoR prediction. In such a system, the interplay between the LLPS boundary and eGT dictates the LoR prediction that is based on the phase diagram (Figure 6).

Figure 6a presents the venetoclax/PVPVA64/water ternary phase diagram at 37 °C with the hydration pathway for 0.5 wt %, 1 wt %, and 2.5 wt % DL ASDs. The API has extremely low solubility in both the polymer and water, such that the solubility line (orange line) runs indistinguishably parallel on the PVPVA64/water axis, implying that all three ASD DLs are already supersaturated in the dry state. Moreover, the system exhibits a very large LLPS region. The predicted large miscibility gap for the ternary system covers almost the entire phase diagram, resulting in an API-rich phase containing almost only venetoclax, along with the polymer-rich phase containing a low venetoclax amount, as indicated by the predicted tie lines. Already, the dry ASD with a DL higher than 5 wt % is thermodynamically predicted to be in the LLPS region and, therefore, is prone to phase separation. Nonetheless, it must be recognized that the system has significantly high *T*_g_ (Figure S1 in the Supplementary Materials) and is, thus, kinetically well-stabilized, which can hinder phase separation in the dry state. Zooming into the phase diagram (Figure 6b) provides insight into the hydration pathway of the different DLs of the ASD. The exact compositions along the hydration pathway in the ternary phase diagram are given in Table 6.

**Table 6:** Venetoclax/PVPVA64/water concentrations along the hydration pathway for 0.5 wt %, 1 wt %, and 2.5 wt % DL venetoclax ASDs at 37 °C (Figure 6a), with the calculated corresponding *T*_g_.

	Water	Venetoclax	PVPVA64	*T*_g_/°C
	0.5 wt % DL ASD			
ASD (dry)	0 wt %	0.5 wt %	99.5 wt %	111.0
eGT	11.2 wt %	0.4 wt %	88.4 wt %	37.0
Solubility limit	APIs supersaturated in the dry state			
Polymer-rich phase at binodal line	20.5 wt %	0.6 wt %	78.9 wt %	−4.4
API-rich phase at binodal line	0.6 wt %	99.4 wt %	0.0 wt %	113.9
	1 wt % DL ASD			
ASD (dry)	0 wt %	1.0 wt %	99.0 wt %	111.1
eGT	11.2 w %	0.9 wt %	87.9 wt %	37.0
Solubility limit	APIs supersaturated in the dry state			
Polymer-rich phase at binodal line	15.6 wt %	0.9 wt %	83.5 wt %	15.8
API-rich phase at binodal line	0.4 wt %	99.6 wt %	0.0 wt %	115.8
	2.5 wt % DL ASD			
ASD (dry)	0 wt %	2.50 wt %	97.50 wt %	111.2
Solubility limit	APIs supersaturated in the dry state			
eGT	LLPS occurs before eGT			37.0
Polymer-rich phase at binodal line	6.1 wt %	2.47 wt %	91.4 wt %	66.5
API-rich phase at binodal line	0.2 wt %	99.8 wt %	0.0 wt %	118.3

The ASD with 0.5 wt % of venetoclax will most likely release into the aqueous medium before LLPS because its hydration pathway meets the binodal line far above eGT. This is evidenced by the high amount of water, 20 wt %, at the binodal line compared to 11 wt % of water content at eGT (Table 6). Thus, phase separation in the gel layer prior to the dissolution of the ASD is unlikely, and a simultaneous API and polymer release is expected. For the 1 wt % DL ASD, the hydration pathway encounters the binodal line at 15 wt % water content, which is very close to the 12 wt % water content at eGT. Thus, LLPS is likely to occur close to the gel layer. The risk of LLPS in the gel layer becomes more obvious with an increasing DL from 1 wt % to 2.5 wt %. For the 2.5 wt % DL ASD, the hydration pathway fully encounters the binodal line before the eGT line, suggesting that LLPS will occur in the gel layer. When LLPS occurs, the PC-SAFT calculated API-rich phase consists of nearly only API with very small amount of water, while the polymer-rich phase consists mostly of polymer and water (Table 6). Hence, the resulting *T*_g_ value of the LLPS phases differs from the initial homogeneous phase. For instance, the calculated *T*_g_ of the formed polymer-rich phase for the 1 wt % DL at the binodal line is 15.8 °C, suggesting high mobility of the polymer-rich phase since the dissolution medium temperature of 37 °C is much higher than the *T*_g_ value. Thus, the high mobility in the polymer-rich phase will allow its release into the aqueous media. In contrast, due to the high *T*_g_ value of the API-rich phase (*T*_g_ = 115.8 °C) and lesser mobility, most of the API will enrich near the gel layer. Situations where LLPS leads to an API-rich phase with a *T*_g_ value higher than the dissolution medium temperature are often referred to as glass liquid phase separation (GLPS) [62]. For the 2.5 wt % DL ASD, according to the LLPS tie lines in Figure 6a, at eGT, the thermodynamic endpoint composition of the polymer-rich phase is estimated to be 12 wt % water and 87 wt % polymer, with a *T*_g_ of 33 °C, while that of the API-rich phase is approximately 0.3 wt % of water and 99 wt % of API, with a *T*_g_ of 117 °C. Due to the high *T*_g_ value of the API-rich phase, most of the APIs can be trapped within the gel layer in the glassy state because the *T*_g_ of this phase is significantly higher than the dissolution medium temperature, 37 °C. According to the lever rule, it is estimated that the API-rich phase constitutes approximately 5% of the total phases at this point. However, considering that when the polymer-rich phase is releasing preferentially, the API-to-polymer ratio will increase; thus, the hydration pathway would curve towards the right over the dissolution process. Thus, the enrichment of venetoclax at the ASD surface over time, coupled with its high *T*_g_ glassy attribute, will cause passivation of the ASD surface, leading to LoR Type II. 

Figure 6c–e shows the METT images of the 0.5 wt %, 1 wt %, and 2.5 wt % DL venetoclax ASDs discs after exposure to water. The multiple small spherical structures seen across the images, especially in the 0.5 wt % and 1 wt % DL ASDs, are trapped air bubbles. Videos of the experimental evolution of the METT ASD disc/water interface are provided in the Supplementary Materials. The images show that the 0.5 wt % DL formulation does not build an LLPS passivating gel layer, unlike the 1 wt % and 2.5 wt % DL formulations. It also appears that the passivated gel layer of the 1 wt % DL formulation is fluid and not rigid, as in the case of the 2.5 wt % DL formulation, because the LLPS occurs closely above eGT. It can also be observed that a light yellowish dispersion is formed in the solution phase away from the interface for the 0.5 wt % DL ASD, which is attributable to the LLPS-driven drug-rich phase droplet formation occurring in the aqueous phase. Thus, applying the ternary phase diagram and concepts presented here, the debate that is often found in the literature as to whether API-rich phase droplet formation occurs in the gel layer or in the solution can be predicted through rigorous thermodynamic modeling [33,35,63]. Overall, the observations from the METT experiments agree very well with the predicted phase behavior of the gel layer, based on the ternary phase diagram, and exactly match the predicted hydration pathways of the ASDs (Table 6), with compositions and *T*_g_s explaining the mobility and occurrence of the resulting phases.

**Figure 7:**
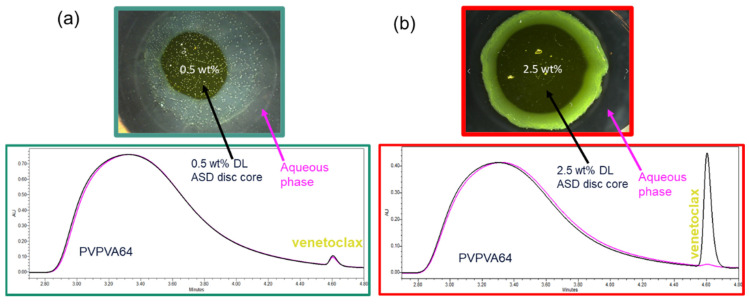
SEC overlay chromatograms of the ASD disc core and the aqueous phase around the ASD after 60 min of dissolution of (**a**) 0.5 wt % DL and (**b**) 2.5 wt % DL venetoclax ASDs.

Since the LoR of venetoclax ASDs occurs at such a low DL, dissolution experiments with the USP 2 apparatus could not be conducted. Nevertheless, the relative amounts of API and polymer released were monitored via the METT experiments. The composition of venetoclax and PVPVA64 in the aqueous phase at the end of the METT experiments was determined via SEC and was then compared with the ASD disc core composition (Figure 7). The supporting information provides all the chromatogram data (Figure S2 in the Supplementary Materials).

**Figure 8:**
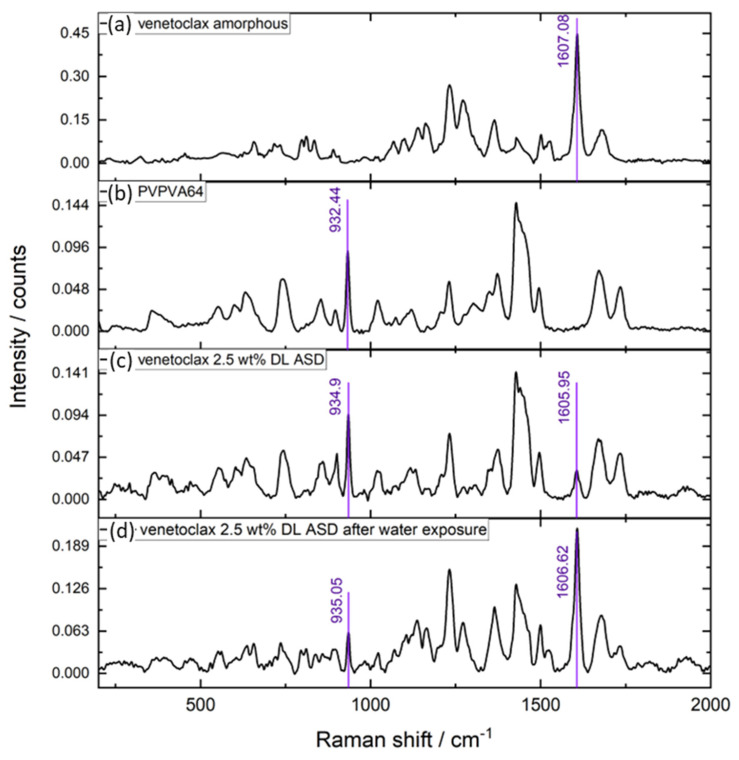
Raman spectra of (**a**) neat amorphous venetoclax, (**b**) neat PVPVA64, (**c**) 2.5 wt % DL venetoclax/PVPVA64 ASD, and (**d**) 2.5 wt % DL venetoclax/PVPVA64 ASD after exposure to water for 240 min at 37 °C.

As shown in Figure 7a, the chromatogram of the aqueous phase of the 0.5 wt % DL ASD is very similar to the chromatogram of the ASD core, suggesting that both the API and polymer were congruently released into the aqueous phase. In contrast, the chromatogram of the aqueous phase of the 2.5 wt % DL of ASD (Figure 7b), compared to the chromatogram of the ASD core, shows only a PVPVA64 peak and an inconspicuous venetoclax peak, thus indicating that the polymer was preferentially released into the aqueous phase while the API was trapped in the gel layer. The enrichment of the API in the gel layer was also directly confirmed via Raman spectroscopy after exposing the 2.5 wt % DL to water, which is shown in Figure 8.

The Raman data show very strong intensification of the API band at ~1607 cm^−1^ after exposure of the ASD to water, while concurrently, the polymer band at ~935 cm^−1^ strongly diminished. Table 7 summarizes the relative amount of API and polymer determined in the aqueous phase for the three formulations after the METT experiments, which confirms that the API and polymer released congruently for the 0.5 wt % and 1 wt % DL ASDs, while at 2.5 wt % DL, LoR occurred, leading to almost only PVPVA64 in the aqueous phase, as predicted using the ternary phase diagram. Thus, for the venetoclax/PVPVA64 ASD system, the limit of congruent (LoC) API and polymer release is 1 wt % DL.

Several attempts have been made in the literature to explain the thermodynamic origin of the typically observed DL-dependent LoR of ASDs. Using a hypothetical ternary phase diagram, Han et al. [35] proposed that LLPS and the eventual passivation of the gel layer occur when spinodal decomposition is triggered at a high drug load below an arbitrarily defined percolation threshold. They argued that this condition leads to the formation of a morphological continuous API-rich phase that passivates the gel layer, while the polymer-rich phase is preferentially released. Recently, the Taylor group [25], also relying on a hypothetical ternary phase diagram, proposed that LoC is defined by the plait point (critical point) wherein the binodal and spinodal lines converge, and assumed that phase separation primarily occurs via spinodal decomposition. To verify these hypotheses, we introduced the PC-SAFT-calculated spinodal line (gray dashed line) in the phase diagram in Figure 6. Based on the hypotheses and assumptions detailed above, phase separation in the gel layer should not be feasible for any of the DL values investigated for the venetoclax/PVPVA64 ASDs since none of the hydration paths runs through the spinodal decomposition region (on the right side of the gray dashed line). However, our experimental data and observations do not support these hypotheses. In our opinion, firstly, these hypotheses are not based on data-driven rigorous thermodynamic modeling and ignore the impact of glass transition, which primarily drives the kinetics of phase separation. Secondly, since dissolution at the ASD/water interface is dynamic, the binodal line is more relevant because it defines compositions at which the phase separation is thermodynamically favorable.

### 4.4. Influence of Temperature on ASD/Water Interfacial Layer

In this section, the influence of the temperature on the interplay between solubility, LLPS, and the glass transition region within the ternary phase diagram was modeled, and the impact on the gel layer was predicted for both naproxen and venetoclax ASD formulations at 50 °C. The 50 °C temperature is physiologically non-relevant but was selected for experimental reasons to investigate the impact of temperature.

#### 4.4.1. LoR Type I at 50 °C: Naproxen/PVPVA64/Water System

**Figure 9:**
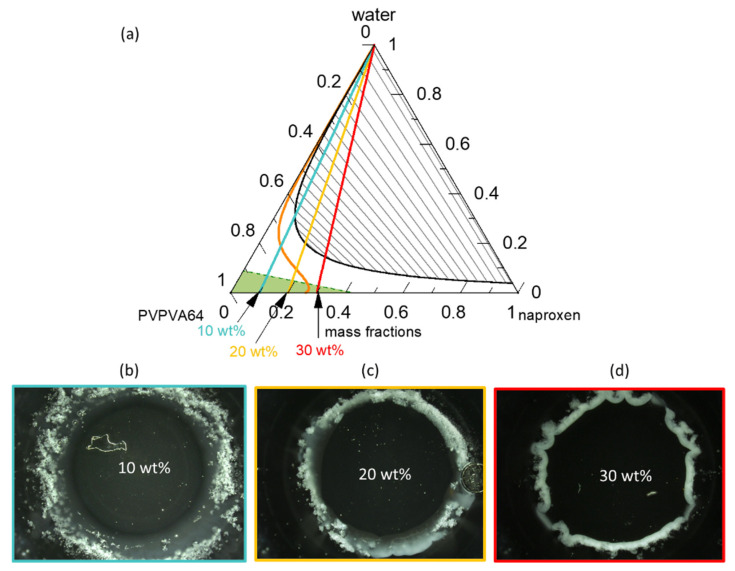
(**a**) Ternary phase diagram of naproxen/PVPVA64/water at 50 °C; the green dashed lines represent the glass transition, the orange lines represent the solubility line, the black line represents the LLPS boundary with gray tie lines, and the blue, yellow, and red straight lines represent the hydration pathways of 10 wt %, 20 wt %, and 30 wt % DL, respectively; METT images after a 30-minute dissolution of a (**b**) 10 wt %, (**c**) 20 wt %, and (**d**) 30 wt % DL ASD disc.

Figure 9 shows the phase diagram and METT images of the naproxen/PVPVA64/water system at 50 °C.

Compared to the phase diagram at 37 °C (Figure 3), the increase in temperature to 50 °C mainly resulted in solubility enhancement and a reduction in the eGT line. The size of the miscibility gap is minimally impacted. To compare the influence of temperature on the API crystallization in the gel layer and the resulting LoR, the same three DL naproxen ASDs were investigated, i.e., 10 wt %, 20 wt %, and 30 wt % DL. The API/water/polymer concentrations along the hydration paths in Figure 9a are listed in Table S2 in the Supplementary Materials. For the 10 wt % DL, at 37 °C, crystallization will not occur in the gel layer but instead in the bulk aqueous layer due to the hydration path intersecting the solubility well above the eGT line at high water concentrations (19 wt %), as seen in the phase diagram. For the 20 wt % and 30 wt % DL ASDs, the temperature effect becomes more apparent. Due to the enhanced API solubility and reduced eGT line at 50 °C compared to 37 °C, API crystallization in the gel layer is predicted for only the 30 wt % DL ASD but not for the 20 wt % DL ASD because the hydration pathway of the latter intersects the solubility line above eGT at 50 °C. The METT image in Figure 9c indeed confirms the prediction for the 20 wt % DL of ASD at 50 °C, compared to the image at 37 °C (Figure 3b). As seen in the 50 °C image, the API crystals formed away from the interface of the eroding ASD disc (which appears as a dark circle in the image), as predicted via the hydration path in Figure 9a. For the 30 wt % DL ASD, crystallization in the gel layer is predicted to occur because the formulation is supersaturated in the dry state at 50 °C. The METT image in Figure 9d confirms the crystallization of the API in the gel layer. However, since the glass region and the eGT are relatively low at 50 °C, the crystallized layer appears fluid compared to the METT image of the same formulation at 37 °C (Figure 3c). The exact compositions along the hydration pathways in the ternary phase diagram are given in the supporting information (Table S2 in the Supplementary Materials).

#### 4.4.2. LoR Type II at 50 °C: Venetoclax/PVPVA64/Water System

**Figure 10:**
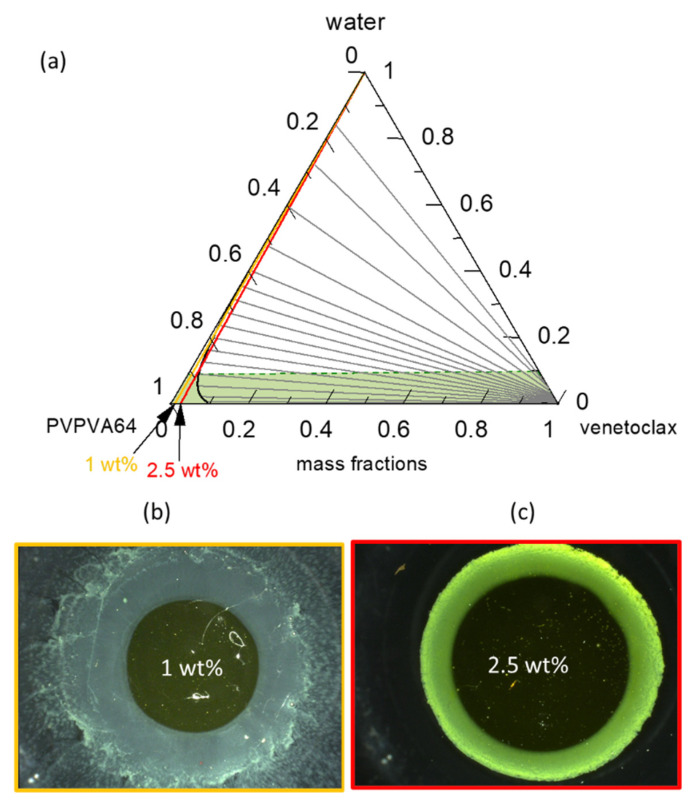
(**a**) Ternary phase diagrams of venetoclax/PVPVA64/water at 50 °C and 0.1 MPa. The green dashed line represents the glass transition, the orange line represents the solubility line, the black line represents the LLPS border with gray tie lines, and the blue, yellow, and red straight lines represent the hydration pathways of a 0.5 wt %, 1 wt %, and 2.5 wt % DL of ASD through the interface concentrations; METT images after 30 min at (**b**) 1 wt % DL and (**c**) 2.5 wt % DL.

Figure 10a shows the modeled venetoclax/PVPVA64/water phase diagram at 50 °C. 

Compared to the phase diagram at 37 °C (Figure 6a), the miscibility gap at 50 °C is smaller. In combination with the slight reduction in the eGT line at this temperature, the miscibility gap reduction indicates that the hydration path of the 1 wt % DL ASD now intersects the binodal line well above eGT when compared to the phase diagram at 37 °C. As a result, no phase separation is observed close to the gel layer, as displayed in the METT image in Figure 10b. For the 2.5 wt % DL of ASD, however, the hydration pathway encounters the binodal line at eGT; hence, phase separation is observed in the gel layer, as seen in the METT image in Figure 10c. Table S3 in the Supplementary Materials provides the API/polymer/water compositions along the hydration pathways in the ternary phase diagram. 

It can be concluded from the modeled temperature effect on solubility, LLPS, and the eGT of the investigated systems that the temperature-dependent release behavior of ASDs can be accurately predicted using the ternary phase diagram.

## 5. Conclusions and Outlook

This work provides thermodynamic modeling insights into the ASD/water interface behavior and release mechanism of PVPV64-based ASDs, based on API/polymer/water ternary phase diagrams. 

With this work, for the first time, we have placed an emphasis on the fundamental understanding of the interfacial surface layers formed when ASD is exposed to water during dissolution, described the possible phase transformations based on the underlying thermodynamics, and, thus, predicted the drug load (DL)-dependent loss of release (LoR). API/polymer/water ternary phase diagrams were modeled using PC-SAFT, combined with the Gordon–Taylor equation. Using the modeled phase diagrams, crystallization and/or liquid-liquid phase separation (LLPS) in the interfacial gel layer or the bulk aqueous phase during dissolution could be predicted quantitatively and were found to be dependent on DL, solubility, miscibility, the amount of water required to reach “escape glass transition” (eGT), and the temperature of the dissolution medium. Depending on the API type, whether a fast or a slow crystallizer from the amorphous phase, it turned out that crystallization is the predominant phase transformation process in the gel layer for the former, while LLPS is the predominant phase transformation in the gel layer for the latter. Crystallization in the gel layer results in the passivation of the ASD surface, which leads to the simultaneous loss of API and polymer release into the dissolution medium. We classified this loss-of-release mechanism as LoR Type I. In contrast, LLPS in the gel layer results in the formation of API-rich and polymer-rich phases, wherein the predicted concentration and glass transition temperature of the phases explain the sudden immobility of the API-rich phase. This passivates the ASD surface, while the polymer-rich phase releases into the dissolution medium, the so-called loss of congruency (LoC) often reported in the literature. We classified this loss-of-release mechanism as LoR Type II. Thus, for the first time, the DL-dependent LoR of ASDs could be systematically classified into two types, depending on the predominant passivation mechanism at the ASD/water interface: API crystallization (LoR Type I) or LLPS (LoR Type II). 

For validation, the DL-dependent release mechanism and LoR for PVPVA64-based naproxen (LoR Type I) and venetoclax (LoR Type II) ASDs were predicted a priori and investigated using microscopy and dissolution experiments at 37 °C. The predictions were found to be in excellent agreement with the experimentally observed gel layer formation, its composition, and the consequent impact on the dissolution of the formulations. Furthermore, the impact of temperature on the ASD/water interface (gel layer) was successfully predicted and experimentally confirmed at 50 °C. 

In summary, the thermodynamic modeling approach and concepts presented here can be used as a predictive early risk assessment tool to categorize API/polymer matrices regarding the complexity of achieving an appropriate DL against LoR. Furthermore, the presented approach supports the identification of the maximum possible DL of a binary API/polymer matrix as a starting point for further ASD formulation design and development experiments.

## Figures and Tables

**Table 1:** The pure components’ melting temperatures, melting enthalpy, and the difference between the solid and liquid heat capacities of the APIs investigated in this work at 0.1 MPa.

Component i	$T_{i}^{SL}/K$	$\Delta h_{i}^{SL}/kJ{mol}^{-1}$	$\Delta c_{p,i}^{SL}/J{mol}^{-1}K^{-1}$
naproxen [7]	429.47	31.50	87
venetoclax	418.15	59.9 *	-

* The melting enthalpy varied between the API lots.

**Table 2:** The pure-component densities and glass-transition temperatures used in this work.

Component i	$\rho_{i}/kgm^{-3}$	$T_{g,i}/K$
naproxen	1250 [46]	265.15 [47]
venetoclax	1340	393.15
PVPVA64	1190 [48]	384.15 [49]
water	1000 [50]	138.00 [51]

**Table 3:** The PC-SAFT pure-component parameters of the components investigated in this work.

Component i	$M_{i}/g{mol}^{-1}$	$m_{i}^{seg}M_{i}^{-1}/molg^{-1}$	$\sigma_{i}/Å$	$u_{i}k_{B}^{-1}/K$	$\varepsilon^{A_{i}B_{i}}k_{B}^{-1}/K$	$\kappa^{A_{i}B_{i}}$	$N_{i}^{assoc}$
naproxen [49]	230.26	0.0352	2.939	229.45	934.2	0.02	2/2
venetoclax	868.44	0.03938	2.546	233.70	0	-	-
PVPVA64 [53]	65,000	0.0372	2.947	205.27	0	0.02	653/653
water [54]	18.015	0.0669	$\sigma_{water}$*	353.95	2425.7	0.0451	1/1

* $\sigma_{water}=2.7927+10.11\;exp(-0.01755T/K)-1.417\;exp(-0.01146T/K)$.

**Table 4:** The PC-SAFT interaction parameters for the mixtures of naproxen, venetoclax, PVPVA64, and water.

Mixture	$k_{ij,m}/K^{-1}$	$k_{ij,b}$
PVPVA64/water [53]	0	−0.1565
naproxen/water [7]	0.000227	−0.0612
naproxen/PVPVA64 [53]	0	−0.0574
venetoclax/water	0	−0.0283
venetoclax/PVPVA64	−0.0000467	0.0075

**Table 5:** Naproxen/PVVPA64/water concentrations along the hydration pathway for 10 wt %, 20 wt %, and 30 wt % DL naproxen ASDs at 37 °C (Figure 3a), with the calculated corresponding *T*_g_.

	Water	Naproxen	PVPVA64	*T*_g_/°C
	10 wt % DL ASD			
ASD (dry)	0 wt %	10 wt %	90.0 wt %	94.6
eGT	9.3 wt %	9.1 wt %	81.6 wt %	37.0
Solubility limit	16.3 wt %	8.4 wt %	75.3 wt %	5.1
Polymer-rich phase at the binodal line	31.0 wt %	6.90 wt %	62.1 wt %	−42.6
API-rich phase at the binodal line	11.0 wt %	35.2 wt %	53.8 wt %	3.2
	20 wt % DL ASD			
ASD (dry)	0 wt %	20.0 wt %	80.0 wt %	79.5
eGT	7.3 wt %	18.5 wt %	74.2 wt %	37.0
Solubility limit	6.1 wt %	18.8 wt %	75.1 wt %	43.2
Polymer-rich phase at the binodal line	22.1 wt %	15.6 wt %	62.3 wt %	−22.1
API-rich phase at the binodal line	15.5 wt %	23.2 wt %	61.3 wt %	−4.1
	30 wt % DL ASD			
ASD (dry)	0 wt %	30.0 wt %	70.0 wt %	65.7
eGT	5.2 wt %	28.4 wt %	66.3 wt %	37.0
Solubility limit	API supersaturated in the dry state			
Polymer-rich phase at the binodal line	24.4 wt %	11.2 wt %	64.4 wt %	−26.3
API-rich phase at the binodal line	14.0 wt %	25.8 wt %	60.2 wt %	−0.7

**Table 7:** Relative venetoclax and PVPVA64 composition in the aqueous phase after the METT experiment.

Formulation	Aqueous Phase	
	Venetoclax/wt %	PVPVA64/wt %
0.5 wt % DL ASD	0.4	99.6
1.0 wt % DL ASD	0.9	99.1
2.5 wt % DL ASD	0.1	99.9

## Data Availability

Data are contained within the article or in the Supplementary Materials.

## Supplementary Materials

The following supporting information can be downloaded at: https://www.mdpi.com/article/10.3390/pharmaceutics15051539/s1, Figure S1: Phase diagram of venetoclax and PVPVA64; Figure S2: SEC overlay chromatograms of the ASD disc core and the aqueous phase around the ASD after 60 min of dissolution; Table S1: venetoclax solubility in water, 2-propanol, and ethyl acetate; Table S2: naproxen/PVPVA64/water concentrations of 10 wt %, 20 wt %, and 30 wt % DL of ASDs at 50 °C; Table S3: venetoclax/PVPVA64/water hydration pathways of 1 wt % and 2.5 wt % DL of ASDs at 50 °C. Videos of ASD discs dissolution at 37 °C and 50 °C. Reference [64] has cited in Supplementary Materials.
