# Supplementary material for: Thermodynamic Modeling of the Amorphous Solid Dispersion-Water Interfacial Layer and Its Impact on the Release Mechanism
Source: Pharmaceutics. 2023 May 19;15(5):1539. doi: 10.3390/pharmaceutics15051539 (PMC10221441; doi:10.3390/pharmaceutics15051539)
Supplement: Supplementary file 1 [file pharmaceutics-15-01539-s001.zip › pharmaceutics-2365277-supplementary.pdf]

pharmaceutics

MDPI

Supplementary Materials: Thermodynamic modeling of the amorphous solid dispersion-water interfacial layer and its impact on the release mechanism

Stefanie Dohrn, Samuel O. Kyeremateng, Esther Bochmann, Ekaterina Sobich, Andrea Wahl, Bernd Liepold, Gabriele Sadowski, and Matthias Degenhardt

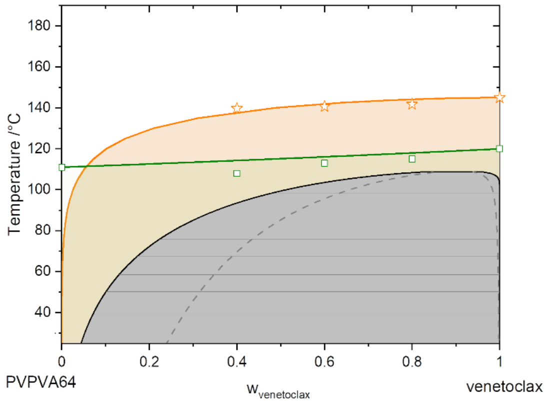

Figure S1. Phase diagram of venetoclax and PVPVA64. The orange line represents the PC-SAFT calculated solubility line, the black and dashed gray lines are PC-SAFT predicted binodal and spinodal lines, respectively, and the green line is the Gordon-Taylor predicted glass-transition line (glassy in the green region below the green line). The mixture is homogeneous in the white region, venetoclax is supersaturated in the matrix in the orange region below the solubility line, and crystallization can occur. Liquid-liquid phase separation (LLPS) and crystallization occur in the gray region. The points represent experimental data, solubility measurements (orange stars), and glass transition temperature (green circles) obtained by DSC measurements based on methods reported by Kyeremateng et al.[1].

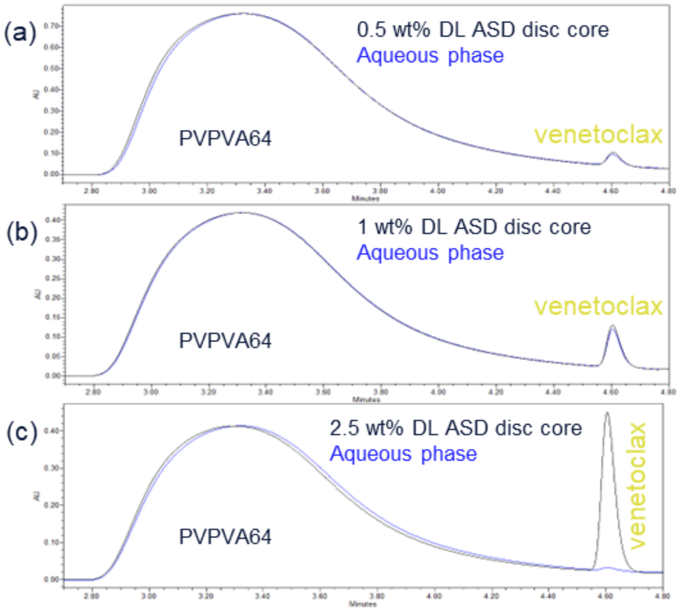

Figure S2. SEC overlay chromatograms of the ASD disc core and the aqueous phase around the ASD after 60 min dissolution of (a) 0.5 wt% DL, (b) 2.5 wt% DL, and (c) 2.5 wt% DL venetoclax ASDs.

Table S1. Venetoclax solubility in water, 2-propanol, and ethyl acetate.

	T / °C	$w_{venetoclax}$	stdev
Water			
	10	$1.129*10^{-08}$	$1.529*10^{-09}$
	25	$1.286*10^{-07}$	$6.739*10^{-08}$
	40	$3.186*10^{-07}$	$2.781*10^{-07}$
2-propanol			
	10	0.00044	0.00007
	25	0.00171	0.00115
	40	0.00701	0.00063
ethyl acetate			
	10	0.00439	0.00111
	25	0.00657	0.00025
	40	0.00988	0.00208

Table S2. Naproxen/PVVPA64/water concentrations along the hydration pathway for 10 wt%, 20 wt%, and 30wt% DL naproxen ASDs at 50 °C and calculated corresponding T_g.

	water	naproxen	PVPVA64	T_g / °C
	10 wt% DL in dry ASD			
ASD	0 wt%	10 wt%	90 wt%	94.6
eGT	6.87 wt%	9.31 wt%	83.81 wt%	50.0
Solubility limit	19.10 wt%	8.09 wt%	72.81 wt%	-5.7
Polymer-rich phase at binodal line	30.84 wt%	6.92 wt%	62.24 wt%	-42.2
API-rich phase at binodal line	11.19 wt%	34.88 wt%	53.93 wt%	2.9
	20 wt% DL in dry ASD			
ASD	0 wt%	20 wt%	80 wt%	79.6
eGT	4.9 wt%	19.0 wt%	76.1 wt%	50.0
Solubility limit	9.5 wt%	18.1 wt%	72.4 wt%	26.3
Polymer-rich phase at binodal line	20.61%	15.88%	63.51%	-17.1
API-rich phase at binodal line	16.76%	20.87%	62.38%	-7.2
	30 wt% DL in dry ASD			
ASD	0 wt%	30 wt%	70 wt%	65.6
eGT	2.7 wt%	29.2%	68.1 wt%	50.0
Solubility limit	API supersaturated in the dry state			
Polymer-rich phase at binodal line	24.54%	11.21%	64.25%	-26.6
API-rich phase at binodal line	14.07%	25.78%	60.15%	-0.9

Table S3. Venetoclax/PVPVA64/water concentrations along hydration pathway for 1 wt% and 2.5 wt% DL venetoclax ASDs at 50 °C, and calculated corresponding T_g.

	water	venetoclax	PVPVA64	T_g / °C
	1 wt% DL in dry ASD			
ASD	0 wt%	1.0 wt%	99.0 wt%	111.1
eGT	8.8 wt%	0.9 wt%	90.3 wt%	50.0
Solubility limit	API supersaturated in the dry state			
Polymer-rich phase at binodal line	20.5 wt%	0.9 wt%	78.6 wt%	-4.4
API-rich phase at binodal line	0.8 wt%	99.2 wt%	0.0 wt%	111.9
	2.5 wt% DL in dry ASD			
ASD	0 wt%	2.5 wt%	97.5 wt%	111.2
eGT	8.8 wt%	2.3 wt%	88.9 wt%	50.0
Solubility limit	API supersaturated in the dry state			

Polymer-rich phase at binodal line	10.0 wt%	2.5 wt%	87.6 wt%	43.6
API-rich phase at binodal line	0.4 wt%	99.6 wt%	0.0 wt%	116.4

Reference

1. Kyeremateng, S.O.; Pudlas, M.; Woehrle, G.H. A fast and reliable empirical approach for estimating solubility of crystalline drugs in polymers for hot melt extrusion formulations. *J. Pharm. Sci.* **2014**, *103*, 2847–2858.
